# Supplementary material for: Distribution of common pipistrelle (Pipistrellus pipistrellus) activity is altered by airflow disruption generated by wind turbines
Source: PLoS One. 2024 May 31;19(5):e0303368. doi: 10.1371/journal.pone.0303368 (PMC11142562; doi:10.1371/journal.pone.0303368)
Supplement: S1 File — SI Statistical analysis—Covariable extraction. SI Statistical analysis—Model weight calculation. S1 Fig. Map showing all 776 wind turbines in the studied counties (within Bretagne and Pays-de-la-Loire regions, western France), the land cover of the area, and the 154 sampled sites. S2 Fig. Boxplot showing that a large gradient of distance from the wind turbine was sampled each night. Horizontal line: median; box: first and third quartiles; whiskers: range; dots: outliers. S3 Fig. Boxplot of the wind incidence angles sampled each night. Various wind incidence angles were sampled each night to obtain a gradient of location around the turbine in relation to wind direction. Horizontal line: median; box: first and third quartiles; whiskers: range; dots: outliers. S4 Fig. Distribution of average wind turbine blade speed rotation per night for the entire dataset (left) and for the three subdatasets (right). S5 Fig. Distribution of the interaction of the tested gradients (wind incidence angle, depending on the distance from the wind turbine) for the entire dataset (left) and for the three subdatasets (right). S6 Fig. Distribution of average wind gusts per night for the entire dataset (left) and for the three subdatasets (right). S7 Fig. Distribution of average wind speed per night for the entire dataset (left) and for the three subdatasets (right). S8 Fig. Number of Pipistrellus pipistrellus each night depending on the average wind speed of the night (i.e. for the three subdatasets). S9 Fig. Candidate models within a ΔAICc < 7 containing (in color) or not (in grey) the variables of interest: distance to wind turbine (blue, on the left), wind incidence angle (orange, in the center), and their interaction (green, on the right). S10 Fig. Estimates, 95% confidence intervals, and p-values for the variables of interest contained in each candidate model within a ΔAICc < 7. S1 Table. Correlation matrix between variables included in the models for all datasets. No variables were corr [file pone.0303368.s001.docx]

**Supporting Information for**

**Airflow disruption by wind turbines alters bat distribution at the landscape scale**

Camille Leroux*^1,2^ (ORCID: 0000-0002-4984-3485), Kévin Barré^1^ (ORCID: 0000-0001-5368-4053), Nicolas Valet^2^, & Christian Kerbiriou^1¶^  (ORCID: 0000-0001-6080-4762), Isabelle Le Viol^1¶^  (ORCID: 0000-0003-3475-5615)

^1^ Centre d'Ecologie et des Sciences de la Conservation (CESCO), Muséum national d'Histoire naturelle, Centre National de la Recherche Scientifique, Sorbonne Université, Station Marine, Concarneau, France.

^2^Auddicé biodiversité– ZAC du Chevalement, Roost-Warendin, France.

^¶^These authors contributed equally to this work.

*Corresponding author:

E-mail: Camille.leroux@edu.mnhn.fr / [camille.leroux@auddice.com](mailto:camille.leroux@auddice.com) (CL)

**Supporting Information**

**[SI Acoustic detection range.](#SI_Acoustic_detection_range)**

**[SI Statistical analysis - Covariable extraction.](#Covariable_extraction)**

[**SI Statistical analysis - Model weight calculation.**](#Model_weight_calculation)

[S1 Fig.](#Fig_S1) Map showing all 776 wind turbines in the studied counties (within Bretagne and Pays-de-la-Loire regions, western France), the land cover of the area, and the 154 sampled sites.

[S2 Fig.](#Fig_S2) Boxplot showing that a large gradient of distance from the wind turbine was sampled each night.

[S3 Fig.](#Fig_S3) Boxplot of the wind incidence angles sampled each night.

[S4 Fig.](#Fig_S4) Distribution of average wind turbine blade speed rotation per night for the entire dataset (left) and for the three subdatasets (right).

[**S5 Fig.**](#Fig_S5) Distribution of the interaction of the tested gradients (wind incidence angle, depending on the distance from the wind turbine) for the entire dataset (left) and for the three subdatasets (right).

[**S6 Fig.**](#Fig_S6) Distribution of average wind gusts per night for the entire dataset (left) and for the three subdatasets (right).

[**S7 Fig.**](#Fig_S7) Distribution of average wind speed per night for the entire dataset (left) and for the three subdatasets (right).

[**S8 Fig.**](#Fig_S8) Number of *Pipistrellus pipistrellus* each night depending on the average wind speed of the night (i.e. for the three subdatasets).

[**S9 Fig**.](#Fig_S9) Candidate models within a ΔAICc < 7 containing (in color) or not (in grey) the variables of interest: distance to wind turbine (blue, on the left), wind incidence angle (orange, in the center), and their interaction (green, on the right).

[**S10 Fig.**](#Fig_S10) Estimates, 95% confidence intervals, and p-values for the variables of interest contained in each candidate model within a ΔAICc < 7.

[**S1 Table.**](#Table_S1) Correlation matrix between variables included in the models for all datasets.

[S2 Table.](#Table_S2) Mean ± standard deviation (min.-max.) of all variables included in the statistical analysis.

[S3 Table.](#Table_S3) AICc and R² of null, full and best models for each dataset.

[S4 Table.](#Table_S4) Estimates ± standard errors and p-values for the variables of interest in the full and best models (GLMMs).

[S5 Table.](#Table_S5) Estimates ± standard errors and p-values for the co variables in the full and best models (GLMMs).

[S6 Table.](#Table_S6) Estimates ± standard errors and p-values (in italics) for the predictors of bat activity for the model resulting from a complementary analysis

Supporting Information Text

SI Acoustic detection range. In agriculture landscape, sound extinction distance can be shorter at the back side of the hedgerow compared to the front side (i.e. microphone side) (Krings et al. 2022). However, this difference has been shown to be noticeable from 30 meters and beyond with Song Meter SM2Bat + model, Wildlife Acoustics, Inc. (Krings et al. 2022). This is outside of the detection range of *P. pipistrellus* which is at the best 30 meters in open habitats (Barataud, 2015). We thus do not expect the location of the microphone to cause any significant acoustic bias depending on the side of the hedgerow in the case of *P. pipistrellus*.

According to the distance of detection of 30 meters for *P. pipistrellus* and the placement of the microphone at about 1.70 above the ground, the recorded activity in this study corresponded to the ground level activity and not to the activity at nacelle or blade height (mean ground clearance height was 49.95 ± 10.91 meters). Thus, in this study, we mainly evaluate the impact of wind turbine wake effect on their foraging activity. These measures at ground height are rather representative of *P. pipistrellus* ecology as the ratio of time spent at nacelle or blade height for this species is expected to be low (about 0.1 according to Roemer et al. 2017).

It must be noted that due to the shape of the wake effect (vertical expansion), the activity measured near the turbine correspond to bats flying lower than the wake effect and thus not exposed to this effect at the time of the recording. However, due to the vertical expansion of the wake effect (see Porté-Agel et al. 2020), we expect to be able to detect bats flying within the wake further from the wind turbine (probably a few hundred meters, depending in particular on the wind speed and the turbine features).

SI Statistical analysis

**Covariable extraction.** Maps of the wind turbines in Bretagne and in Pays-de-la-Loire were extracted from the Geobretagne and sigloire catalogs (2020), respectively. We calculated the hedgerow density in a buffer zone of 1500m (in line with the maximum sampling distance from wind turbines) based on the BD Haie map (IGN, 2020), and the distance from the nearest watercourse or water body based on the French hydrographic reference map BD Topage (IGN and OFB, 2019). All weather data were manually extracted from meteociel (meteociel.fr) at a temporal resolution of one hour. Finally, data on wind turbine rotor diameter and rotation per minute at a temporal resolution of 10 minutes were provided by wind farm operators. All calculations of hedgerow density, distance from water and from the nearest wind turbine, average wind speed and temperature of the night as well as average blade rotation speed of the night were performed in R software (RStudio, 2022). The prevailing wind of the night was defined as the first mode of the wind direction distribution during the night and was computed with the mlv function (meanshift method; r package modeest). We visually checked wind direction distribution for each site-night to ensure that there was not a second mode too different from the first; this was never the case.

**Model weight calculation.**

Model weight = $1-\frac{\left( 10-N \right)}{10}$

With 10 the total number of hours in a night

With N the number of hours in which the wind direction was close (± 22.5°) to the prevailing wind direction (mode) of the night. We choose an interval of 45° around the prevailing wind direction to ensure that its upper and lower limits would not share more than two main wind directions (N, NE, E, SE, S, SW, W, NW) around the prevailing wind direction.


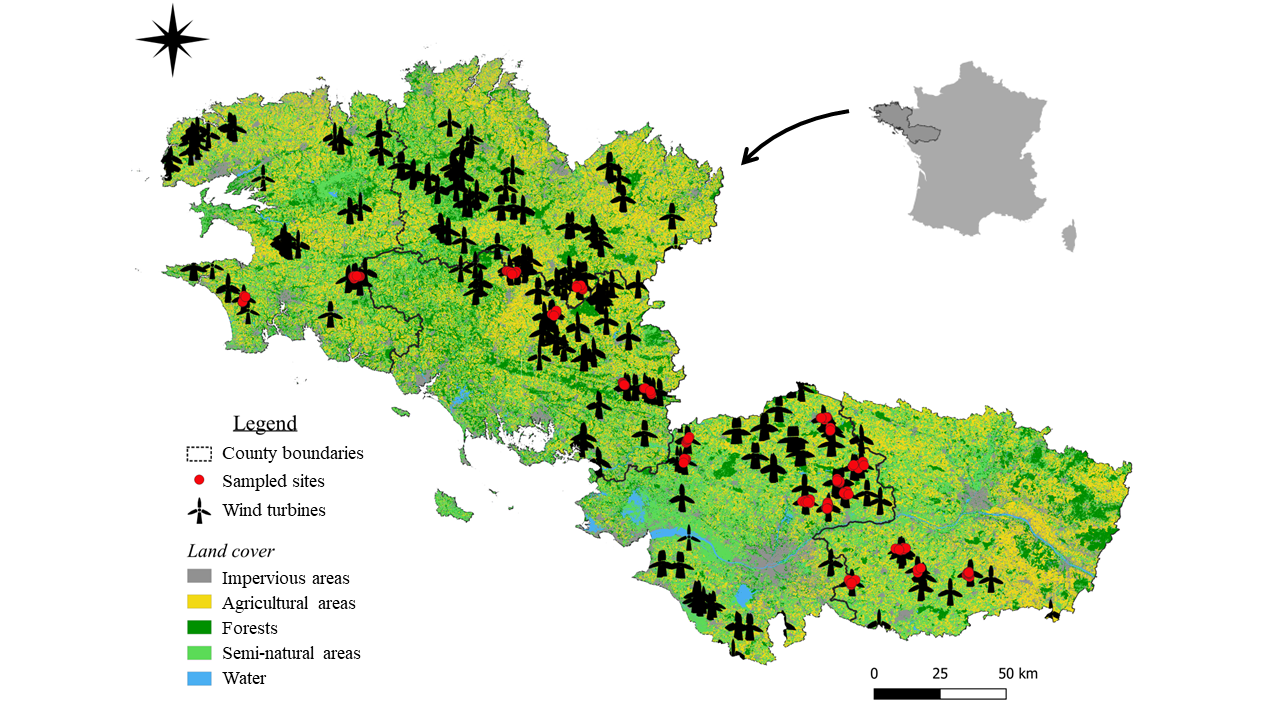


Fig. S1. Map showing all 776 wind turbines in the studied counties (within Bretagne and Pays-de-la-Loire regions, western France), the land cover of the area, and the 154 sampled sites.


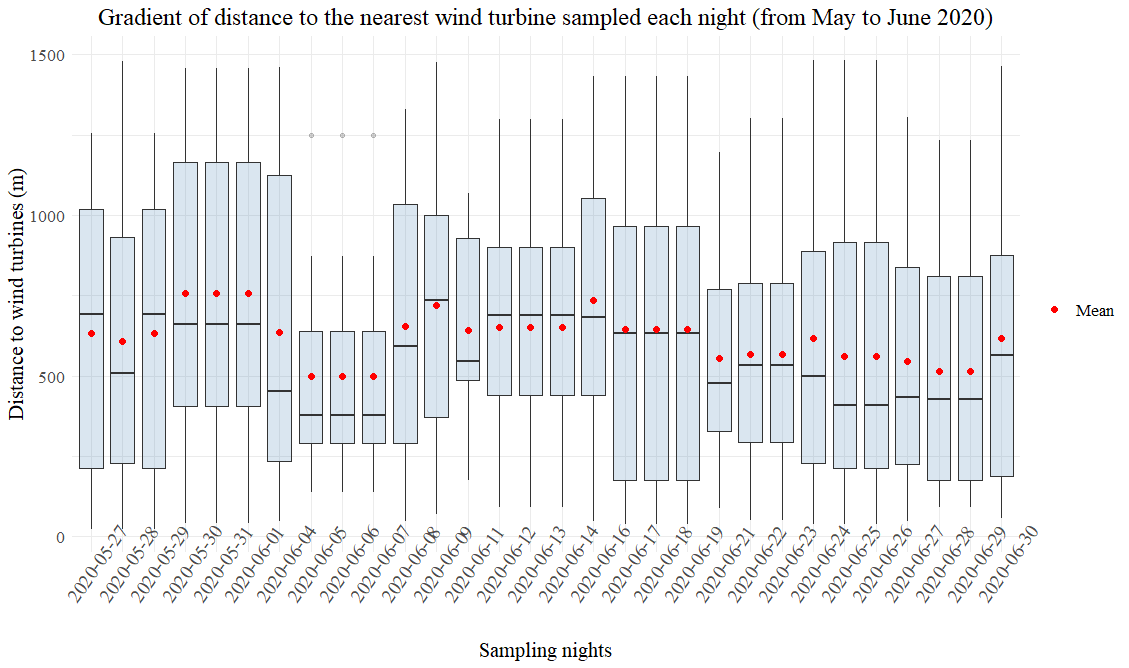


Fig. S2. Boxplot showing that a large gradient of distance from the wind turbine was sampled each night. Horizontal line: median; box: first and third quartiles; whiskers: range; dots: outliers.


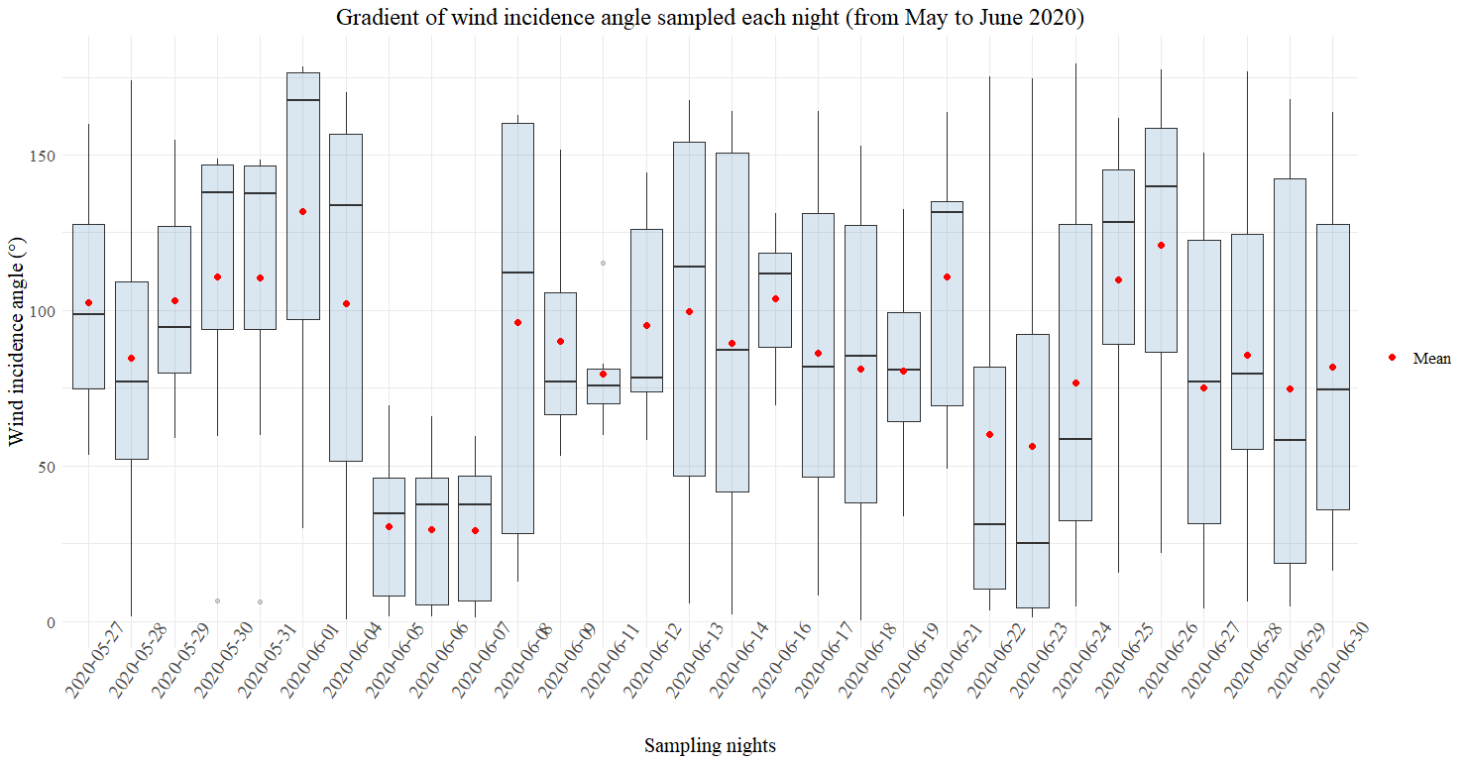


Fig. S3. Boxplot of the wind incidence angles sampled each night. Various wind incidence angles were sampled each night to obtain a gradient of location around the turbine in relation to wind direction. Horizontal line: median; box: first and third quartiles; whiskers: range; dots: outliers.


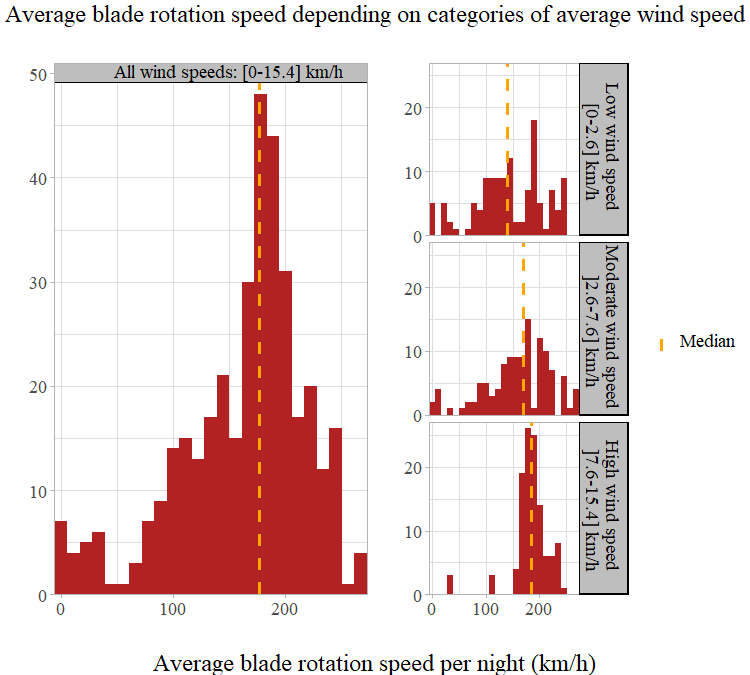


Fig. S4. Distribution of average wind turbine blade speed rotation per night for the entire dataset (left) and for the three subdatasets (right).


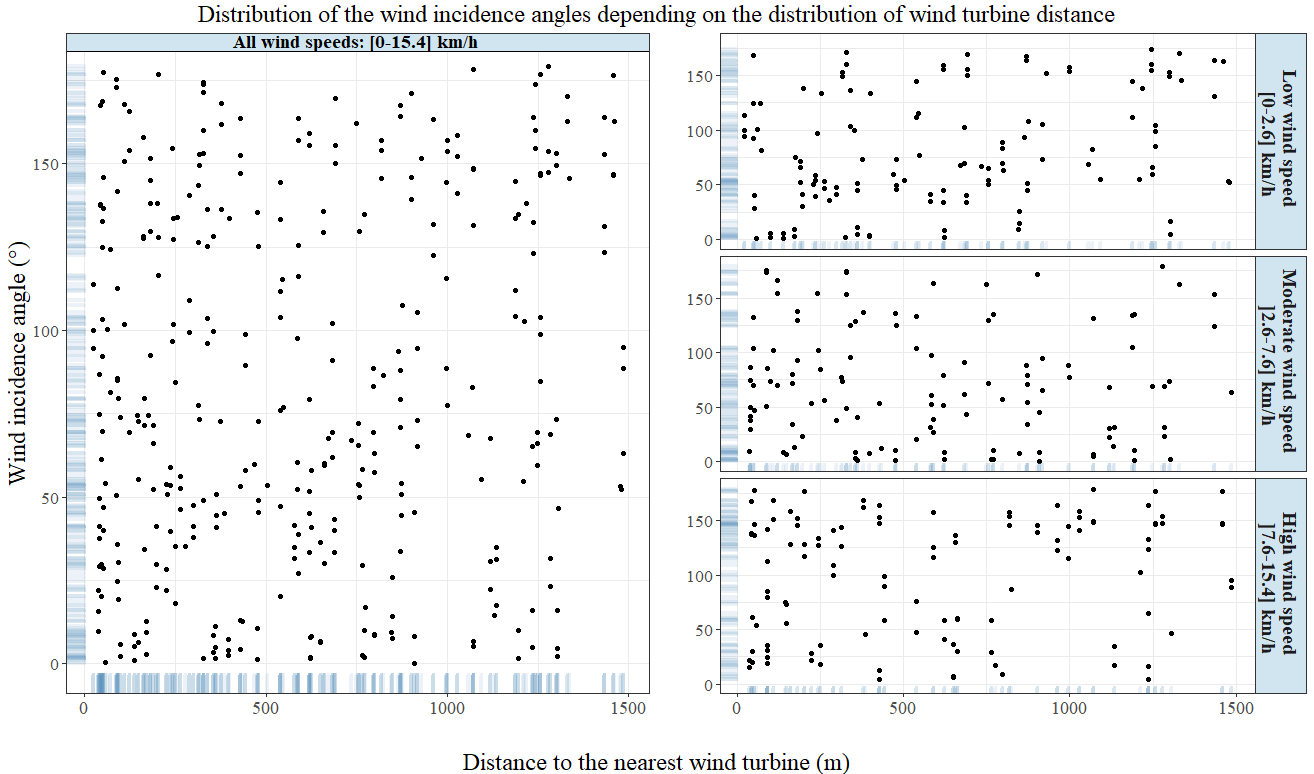


Fig. S5. Distribution of the interaction of the tested gradients (wind incidence angle, depending on the distance from the wind turbine) for the entire dataset (left) and for the three subdatasets (right).


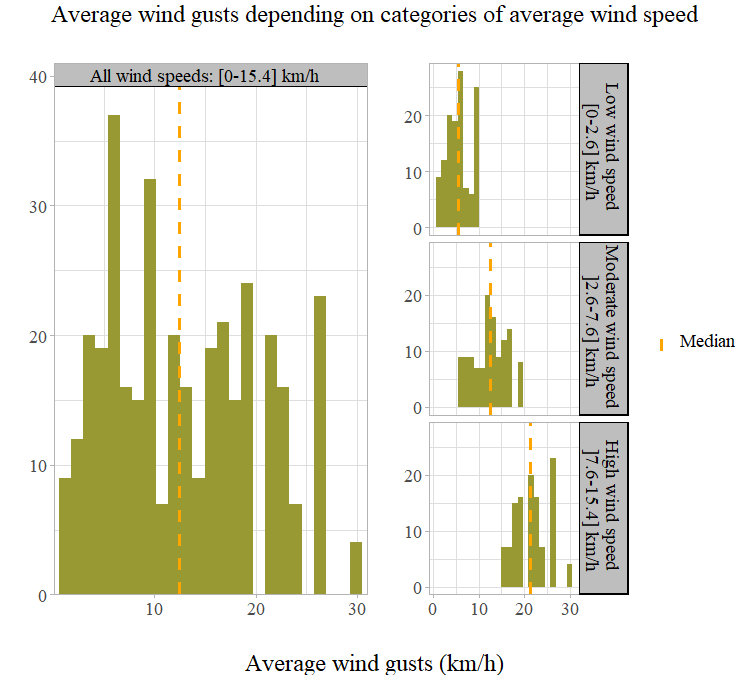


Fig. S6. Distribution of average wind gusts per night for the entire dataset (left) and for the three subdatasets (right).


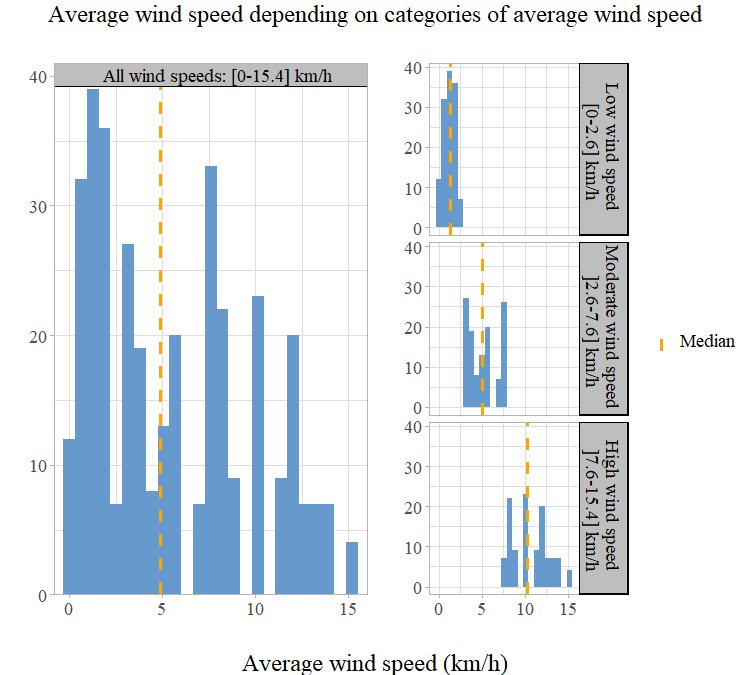


Fig. S7. Distribution of average wind speed per night for the entire dataset (left) and for the three subdatasets (right).


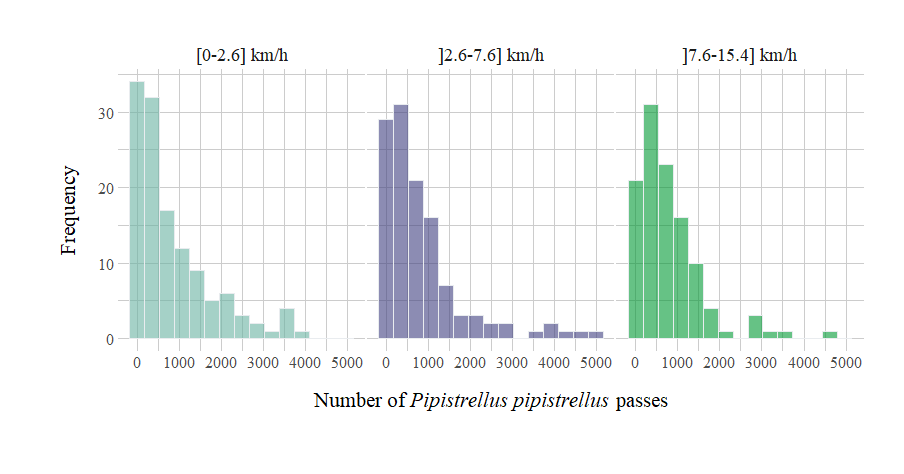


**Fig. S8.** Number of *Pipistrellus pipistrellus* each night depending on the average wind speed of the night (i.e. for the three subdatasets).


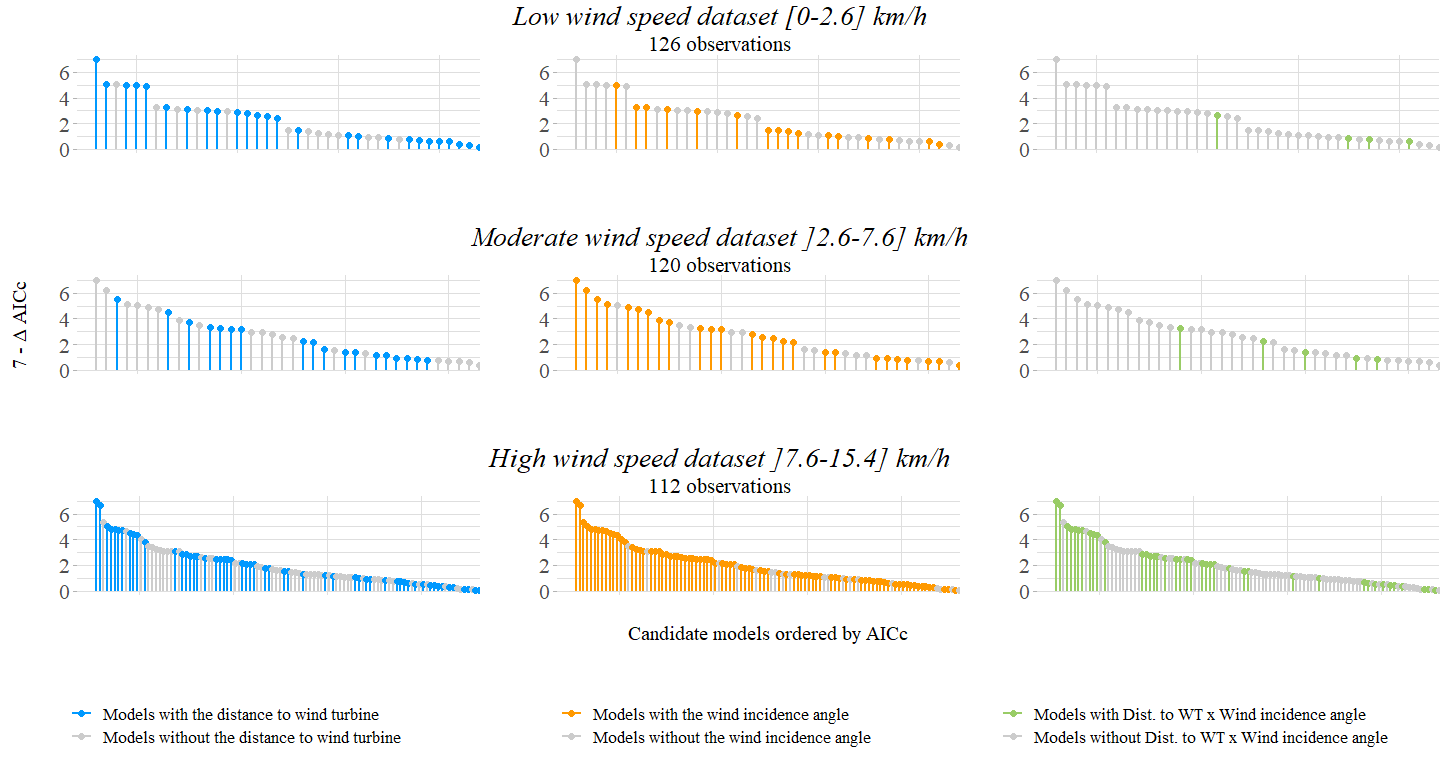


Fig. S9. Candidate models within a ΔAICc < 7 containing (in color) or not (in grey) the variables of interest: distance to wind turbine (blue, on the left), wind incidence angle (orange, in the center), and their interaction (green, on the right).


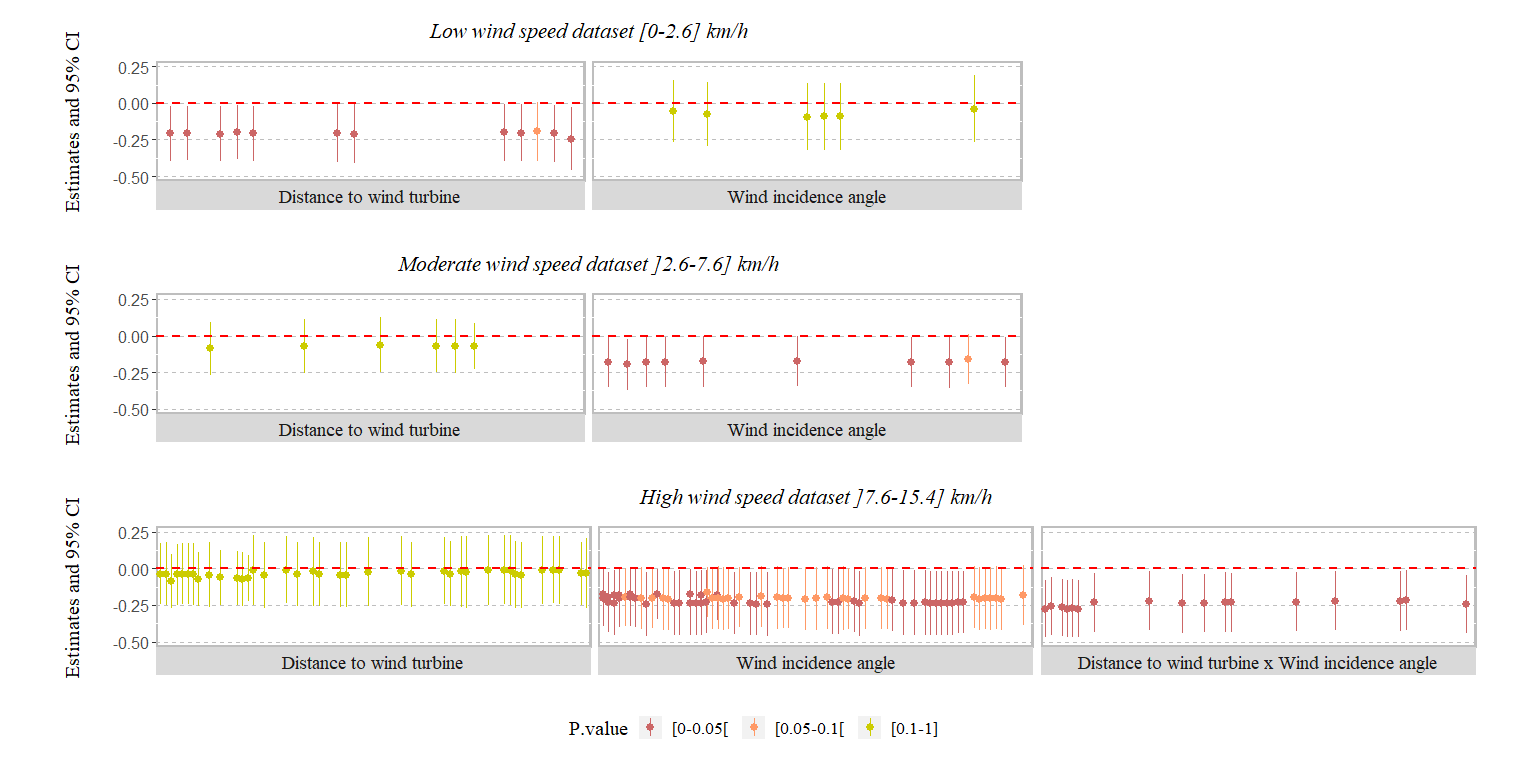
Fig. S10. Estimates, 95% confidence intervals, and p-values for the variables of interest contained in each candidate model within a ΔAICc < 7.

Table S1. Correlation matrix between variables included in the models for all datasets. No variables were correlated (r < |0.7|). Besides this correlation check, we checked for potential collinearity problems in the full models using the Variance Inflation Factor (VIF) before modelling (R package performance). WT = wind turbine.

| **All wind speeds [0 ;15.4] km/h** | Dist. to WT | Average wind | Angle of incidence | Rotor diameter | Blade speed | Hedgerow length | Dist. to water | Average temperature |
| --- | --- | --- | --- | --- | --- | --- | --- | --- |
| Dist. to WT | 1 |  |  |  |  |  |  |  |
| Average wind | -0.05 | 1 |  |  |  |  |  |  |
| Wind incidence angle | 0.07 | 0.17 | 1 |  |  |  |  |  |
| Rotor diameter | -0.03 | -0.19 | 0.03 | 1 |  |  |  |  |
| Blade speed | 0.01 | 0.35 | 0.08 | -0.07 | 1 |  |  |  |
| Hedgerow length | 0.03 | 0.30 | 0.01 | -0.02 | 0.19 | 1 |  |  |
| Dist. to water | -0.08 | -0.06 | 0.09 | 0.05 | 0.04 | -0.03 | 1 |  |
| Average temperature | -0.04 | 0.47 | 0.10 | -0.18 | 0.09 | 0.16 | -0.15 | 1 |

| **(1)** | **Wind speed [0 ;2.6] km/h** | Dist. to WT | Average wind | Angle of incidence | Rotor diameter | Blade speed | Hedgerow length | Dist. to water | Average temperature |
| --- | --- | --- | --- | --- | --- | --- | --- | --- | --- |
| Dist. to WT | | 1 |  |  |  |  |  |  |  |
| Average wind | | -0.03 | 1 |  |  |  |  |  |  |
| Wind incidence angle | | 0.20 | 0.20 | 1 |  |  |  |  |  |
| Rotor diameter | | -0.05 | -0.11 | -0.03 | 1 |  |  |  |  |
| Blade speed | | 0.00 | 0.05 | 0.17 | -0.23 | 1 |  |  |  |
| Hedgerow length | | 0.04 | -0.13 | -0.09 | -0.01 | 0.12 | 1 |  |  |
| Dist. to water | | -0.06 | -0.03 | -0.02 | -0.1 | 0.21 | -0.31 | 1 |  |
| Average temperature | | -0.01 | 0.41 | 0.04 | -0.21 | -0.25 | 0.14 | -0.16 | 1 |

| **(2)** | **Wind speed ]2.6 ; 7.6] km/h** | Dist. to WT | Average wind | Angle of incidence | Rotor diameter | Blade speed | Hedgerow length | Dist. To water | Average temperature |
| --- | --- | --- | --- | --- | --- | --- | --- | --- | --- |
| Dist. to WT | | 1 |  |  |  |  |  |  |  |
| Average wind | | -0.01 | 1 |  |  |  |  |  |  |
| Wind incidence angle | | -0.07 | -0.04 | 1 |  |  |  |  |  |
| Rotor diameter | | 0.00 | 0.04 | 0.06 | 1 |  |  |  |  |
| Blade speed | | 0.04 | 0.28 | -0.11 | 0.04 | 1 |  |  |  |
| Hedgerow length | | 0.06 | 0.15 | -0.17 | -0.07 | -0.02 | 1 |  |  |
| Dist. to water | | -0.22 | 0.08 | 0.18 | 0.26 | 0.02 | 0.18 | 1 |  |
| Average temperature | | 0.00 | 0.08 | -0.01 | -0.16 | 0.13 | 0.01 | -0.03 | 1 |

| **(3)** | **Wind speed ]7.6 ; 15.4] km/h** | Dist. to WT | Average wind | Angle of incidence | Rotor diameter | Blade speed | Hedgerows density | Dist. to water | Average temperature |
| --- | --- | --- | --- | --- | --- | --- | --- | --- | --- |
| Dist. to WT | | 1 |  |  |  |  |  |  |  |
| Average wind | | 0.05 | 1 |  |  |  |  |  |  |
| Wind incidence angle | | 0.11 | 0.12 | 1 |  |  |  |  |  |
| Rotor diameter | | -0.09 | -0.54 | 0.02 | 1 |  |  |  |  |
| Blade speed | | 0.13 | 0.17 | 0.06 | 0.32 | 1 |  |  |  |
| Hedgerow density | | 0.05 | 0.00 | 0.03 | -0.02 | 0.40 | 1 |  |  |
| Dist. to water | | 0.03 | 0.07 | 0.06 | -0.29 | -0.1 | -0.01 | 1 |  |
| Average temperature | | -0.04 | -0.19 | 0.09 | 0.01 | -0.32 | -0.20 | -0.26 | 1 |

Table S2. Mean ± standard deviation (min.-max.) of all variables included in the statistical analysis.

| Variables | All wind speeds | [0-2.6] km/h | ]2.6-7.6] km/h | ]7.6-15.4] km/h |
| --- | --- | --- | --- | --- |
| Tested variables: |  |  |  |  |
| Distance to the nearest wind turbine (m) | 604.12 ± 427.04  (23.21-1484.00) | 625.86 ± 421.20  (23.21-1479.12) | 592.55 ± 416.47  (37.83-1484.00) | 592.05 ± 447.17  (37.83-1484.00) |
| Wind incidence angle (°) | 83.55 ± 54.32  (0.16-179.24) | 80.91 ± 52.08  (0.53-173.92) | 71.39 ± 52.48  (0.16-179.24) | 99.53 ± 55.29  (4.20-178.39) |
|  |  |  |  |  |
| Covariables: |  |  |  |  |
| Blade rotation speed (km/h) | 163.5 ± 56.62  (0.00-267.70) | 144.4 ± 64.59  (0.00-250.50) | 160.60 ± 60.98  (2.99-267.72) | 188.20 ± 24.21  (112.60-240.30) |
| Rotor diameter (m) | 84.39 ± 10.75  (70.00-114.00) | 87.65 ± 10.44  (70.00-109.80) | 81.17 ± 10.85  (70.00-114) | 84.17 ± 9.97  (70.00-100.00) |
| Hedgerow length in a 1500m buffer (m) | 48671.00 ± 12977.50  (19825.00-81560) | 46521 ± 14239.82  (19825.00-81560.00) | 44066.00 ± 11327.52  (24646.00-74507.00) | 56024.00 ± 9644.68  (29635.00-74507.00) |
| Distance to water bodies/courses (m) | 288.01 ± 189.42  (0.27-1119.66) | 358.16 ± 239.20  (0.27-1119.66) | 235.64 ± 144.63  (0.73-602.76) | 265.19 ± 139.50  (1.83-538.98) |
| Average wind speed | 5.53 ± 4.19  (0.20-15.40) | 1.252 ± 0.68  (0.20-2.60) | 5.11 ± 1.65  (3.00-7.60) | 10.78 ± 2.09  (7.80-15.40) |
| Average temperature | 14.41 ± 2.65  (8.94-22.06) | 12.86 ± 2.36  (8.94-19.71) | 14.47 ± 2.68  (12.21-22.06) | 16.10 ± 1.77  (12.80 ± 19.60) |

**Table S3.** AICc and R² of null, full and best models for each dataset. Note that the full model of the low wind speed dataset ([0-2.6] km/h) presented convergence problems and the null model of the moderate wind speed dataset suffered from singularity. We therefore could not extract the AICc and the conditional R², respectively, for these models. The model weight detailed in SI Appendix, Statistical Analysis, was included in all the models. Also note that we included a random effect on the night when running models on the full dataset to control for inter-night variations in bat activity. We did not include it on the models performed on the subdatasets as it explained very little deviance part and led to convergence problems. WT= wind turbine.

| **Wind speed** | **Model** | | **AICc** | **R^2^** |
| --- | --- | --- | --- | --- |
| All wind speeds | Null : | 1 + (1\|night) + (1\|site) | 3635.80 | C : 0.55  M : 0.00 |
|  | Full : | Log(Dist. WT + 1) * Wind incidence angle + Rotor diameter + Blade speed + Hedgerow length + Log(Dist. water + 1) + Mean temperature + Mean wind speed + (1\|night) + (1\|site) | 3620.79 | C : 0.57  M : 0.18 |
|  | Best : | Log(Dist. WT + 1) * Wind incidence angle + Rotor diameter + Blade speed + Mean wind speed + Mean temperature + (1\|night) + (1\|site) | 3619.90 | C : 0.57  M : 0.17 |
| [0-2.6] km/h | Null : | 1 + (1\|site) | 988.49 | C : 0.258  M : 0.000 |
|  | Full : | Log(Dist. WT + 1) * Wind incidence angle + Rotor diameter + Blade speed + Hedgerow length + Log(Dist. water + 1) + Mean temperature + Mean wind speed + (1\|site) | / | C : 0.99  M : 0.01 |
|  | Best : | Log(Dist. WT + 1) + Rotor diameter + Mean temperature + Dist. water + (1\|site) | 967.18 | C : 0.46  M : 0.39 |
| ]2.6-7.6] km/h | Null : | 1 + (1\|site) | 1364.32 | C : NA  M : 0.000 |
|  | Full : | Log(Dist. WT + 1) * Wind incidence angle + Rotor diameter + Blade speed + Hedgerow length + Log(Dist. water + 1) + Mean temperature + Mean wind speed + (1\|site) | 1350.77 | C : 0.34  M : 0.32 |
|  | Best : | Wind incidence angle + Rotor diameter + Mean wind speed + Blade speed + (1\|site) | 1342.11 | C : 0.50  M : 0.34 |
| ]7.6-15.4] km/h | Null : | 1 + (1\|site) | 1307.65 | C : 0.593  M : 0.000 |
|  | Full : | Log(Dist. WT + 1) * Wind incidence angle + Rotor diameter + Blade speed + Hedgerow length + Log(Dist. water + 1) + Mean temperature + Mean wind speed + (1\|site) | 1311.65 | C : 0.61  M : 0.19 |
|  | Best : | Log(Dist. WT + 1) * Wind incidence angle + Mean temperature + (1\|site) | 1302.79 | C : 0.57  M : 0.17 |

Table S4. Estimates ± standard errors and p-values for the variables of interest in the full and best models (GLMMs).

| **Wind speed (km/h)** | **Model** | **Intercept** | **Log(Dist. WT + 1)** | **Wind incidence angle** | **Log(Dist. WT + 1)*Wind incidence angle** |
| --- | --- | --- | --- | --- | --- |
| All wind speeds | Full | 6.421 ± 0.106  **< 0.001** | -0.078 ± 0.065  0.231 | -0.171 ± 0.062  **0.006** | -0.129 ± 0.062  **0.039** |
|  | Best | 6.448 ± 0.107  **< 0.001** | -0.095 ± 0.066  0.147 | -0.161 ± 0.062  **0.009** | -0.125 ± 0.063  **0.047** |
| [0-2.6] | Full | *This model does not converge.* | | | |
|  | Best | 6.472 ± 0.168  **< 0.001** | -0.208 ± 0.096  **0.030** | / | / |
| ]2.6-7.6] | Full | 6.631 ± 0.165  **< 0.001** | -0.077 ± 0.088  0.383 | -0.207 ± 0.089  **0.020** | -0.016 ± 0.095  0.866 |
|  | Best | 6.545 ± 0.145  **< 0.001** | / | -0.177 ± 0.087  **0.043** | / |
| ]7.6-15.4] | Full | 6.465 ± 0.118  **< 0.001** | -0.031 ± 0.108  0.772 | -0.224 ± 0.103  **0.030** | -0.249 ± 0.101  **0.014** |
|  | Best | 6.488 ± 0.116  **< 0.001** | -0.036 ± 0.107  0.733 | -0.199 ± 0.100  **0.047** | -0.274 ± 0.100  **0.006** |

Table S5. Estimates ± standard errors and p-values for the covariables in the full and best models (GLMMs).

| **Wind speed (km/h)** | | **Model** | **Blade rotation speed** | | | **Rotor diameter** | | **Hedgerows length in a 1500m buffer** | | **Average wind speed** | | **Average temperature** | | **Log(Distance to water + 1)** | |
| --- | --- | --- | --- | --- | --- | --- | --- | --- | --- | --- | --- | --- | --- | --- | --- |
| All wind speeds | Full | | | 0.182 ± 0.081  **0.025** | | | -0.245 ± 0.084  **0.004** | | -0.095 ± 0.079  0.227 | | -0.154 ± 0.099  0.118 | | 0.229 ± 0.096  **0.017** | | 0.097 ± 0.071  0.170 |
|  | Best | | | 0.187 ± 0.082  **0.023** | | | -0.262 ± 0.085  **0.002** | | / | | -0.161 ± 0.098  0.100 | | 0.188 ± 0.093  **0.044** | | / |
| [0-2.6] | Full | | | | *This model does not converge.* | | | | | | | | | | |
|  | Best | | | **/** | | | -0.392 ± 0.125  **0.002** | | / | | / | | 0.258 ± 0.092  **0.005** | | 0.414 ± 0.144  **0.004** |
| ]2.6-7.6] | Full | | | 0.343 ± 0.140  **0.014** | | | -0.251 ± 0.109  **0.022** | | -0.105 ± 0.099  0.286 | | -0.325 ± 0.092  **<0.001** | | -0.001 ± 0.105  0.992 | | 0.127 ± 0.099  0.199 |
|  | Best | | | 0.420 ± 0.115  **<0.001** | | | -0.252 ± 0.096  **0.009** | | / | | -0.350 ± 0.086  **<0.001** | | / | | / |
| ]7.6-15.4] | Full | | | -0.081 ± 0.112  0.468 | | | 0.060 ± 0.137  0.660 | | 0.110 ± 0.110  0.319 | | 0.151 ± 0.092  0.102 | | 0.212 ± 0.089  **0.017** | | -0.039 ± 0.116  0.739 |
|  | Best | | | **/** | | | / | | **/** | | **/** | | 0.197 ± 0.083  **0.017** | | / |

**Table S6**. Estimates ± standard errors and p-values (*in* *italics*) for the predictors of bat activity for the model resulting from a complementary analysis: we split the high wind-speed dataset into two subdatasets around the median of the wind incidence angle (119.5°) to test for the effect of the distance from wind turbine windward of and leeward of the turbine. We tested this effect on the best model (GLMM) of the high wind-speed dataset from which we removed the wind incidence angle term.

|  | **High wind speed**  **]7.6-15.4] km/h** | |
| --- | --- | --- |
|  | **Winward the turbine**  **(α ≤ 119.5; n=56)** | **Leeward the turbine (α > 119.5; n=56)** |
| Log(Distance from wind turbine (m) + 1) | 0.301 ± 0.136  ***0.027*** | -0.419 ± 0.162  ***0.010*** |
| Mean temperature (C°) | 0.242 ± 0.114  ***0.034*** | 0.197 ± 0.109  *0.071* |
