# Supplementary material for: Distribution of common pipistrelle (Pipistrellus pipistrellus) activity is altered by airflow disruption generated by wind turbines
Source: PLoS One. 2024 May 31;19(5):e0303368. doi: 10.1371/journal.pone.0303368 (PMC11142562; doi:10.1371/journal.pone.0303368)
Supplement: Attachment — Submitted filename: Response to Reviewers.docx [file pone.0303368.s002.docx]

**Journal requirements:**

When submitting your revision, we need you to address these additional requirements.

1. Please ensure that your manuscript meets PLOS ONE's style requirements, including those for file naming. The PLOS ONE style templates can be found at

https://journals.plos.org/plosone/s/file?id=wjVg/PLOSOne_formatting_sample_main_body.pdf and

<https://journals.plos.org/plosone/s/file?id=ba62/PLOSOne_formatting_sample_title_authors_affiliations.pdf>

>> We modified title font size, affilation format, as well as citation format. We also added the list of supporting information at the end of the manuscript.

2. Thank you for stating the following financial disclosure:

“We thank the Association Nationale de la Recherche et de la Technologie (Grant No. 2019/1566) and Auddicé biodiversité for funding this research; IN2P3 Computing Centre and PCIA-MNHN for providing computing and storage facilities to process and archive in the long term all the bat recordings used in this study; Yves and Didier Bas for their help with the archiving; wind farm developers for funding part of the bat recorders; wind farm developers, operators and owners, for providing us with information on features of the wind turbines and blade rotation speeds for the nights on which we sampled; Nancy Jennings for the editing service; and Jérémy Prouff, Anne-Laure Brissard and Jean-Noël Caron for helping with the figures. Kévin Barré was funded by the Agence de la transition écologique (ADEME), Christian Kerbiriou by Sorbonne University, and Isabelle Le Viol by the french National Museum of Natural History (MNHN). »

Please state what role the funders took in the study.  If the funders had no role, please state: "The funders had no role in study design, data collection and analysis, decision to publish, or preparation of the manuscript."

If this statement is not correct you must amend it as needed.

Please include this amended Role of Funder statement in your cover letter; we will change the online submission form on your behalf. >> We included the amended Funding Statement in the cover letter.

3. Thank you for stating the following in the Acknowledgments Section of your manuscript:

“We thank the Association Nationale de la Recherche et de la Technologie (Grant No. 2019/1566) and Auddicé biodiversité for funding this research; IN2P3 Computing Centre and PCIA-MNHN for providing computing and storage facilities to process and archive in the long term all the bat recordings used in this study; Yves and Didier Bas for their help with the archiving; wind farm developers for funding part of the bat recorders; wind farm developers, operators and owners, for providing us with information on features of the wind turbines and blade rotation speeds for the nights on which we sampled; Nancy Jennings for the editing service; and Jérémy Prouff, Anne-Laure Brissard and Jean-Noël Caron for helping with the figures. Kévin Barré was funded by the Agence de la transition écologique (ADEME), Christian Kerbiriou by Sorbonne University, and Isabelle Le Viol by the french National Museum of Natural History (MNHN).”

We note that you have provided funding information that is not currently declared in your Funding Statement. However, funding information should not appear in the Acknowledgments section or other areas of your manuscript. We will only publish funding information present in the Funding Statement section of the online submission form. >> We removed funding information from the manuscript.

Please remove any funding-related text from the manuscript and let us know how you would like to update your Funding Statement. Currently, your Funding Statement reads as follows:

“We thank the Association Nationale de la Recherche et de la Technologie (Grant No. 2019/1566) and Auddicé biodiversité for funding this research; IN2P3 Computing Centre and PCIA-MNHN for providing computing and storage facilities to process and archive in the long term all the bat recordings used in this study; Yves and Didier Bas for their help with the archiving; wind farm developers for funding part of the bat recorders; wind farm developers, operators and owners, for providing us with information on features of the wind turbines and blade rotation speeds for the nights on which we sampled; Nancy Jennings for the editing service; and Jérémy Prouff, Anne-Laure Brissard and Jean-Noël Caron for helping with the figures. Kévin Barré was funded by the Agence de la transition écologique (ADEME), Christian Kerbiriou by Sorbonne University, and Isabelle Le Viol by the french National Museum of Natural History (MNHN).”

Please include your amended statements within your cover letter; we will change the online submission form on your behalf. >> We included the amended Funding Statement and Acknowledgements in the cover letter. We also amended directly the Acknowledgement statement in the manuscript.

4. We note that Figure 1 in your submission contain copyrighted images. All PLOS content is published under the Creative Commons Attribution License (CC BY 4.0), which means that the manuscript, images, and Supporting Information files will be freely available online, and any third party is permitted to access, download, copy, distribute, and use these materials in any way, even commercially, with proper attribution. For more information, see our copyright guidelines: http://journals.plos.org/plosone/s/licenses-and-copyright.

We require you to either (1) present written permission from the copyright holder to publish these figures specifically under the CC BY 4.0 license, or (2) remove the figures from your submission:

1. You may seek permission from the original copyright holder of Figure 1 to publish the content specifically under the CC BY 4.0 license.

We recommend that you contact the original copyright holder with the Content Permission Form (http://journals.plos.org/plosone/s/file?id=7c09/content-permission-form.pdf) and the following text:

“I request permission for the open-access journal PLOS ONE to publish XXX under the Creative Commons Attribution License (CCAL) CC BY 4.0 (http://creativecommons.org/licenses/by/4.0/). Please be aware that this license allows unrestricted use and distribution, even commercially, by third parties. Please reply and provide explicit written permission to publish XXX under a CC BY license and complete the attached form.”

Please upload the completed Content Permission Form or other proof of granted permissions as an "Other" file with your submission.

 In the figure caption of the copyrighted figure, please include the following text: “Reprinted from [ref] under a CC BY license, with permission from [name of publisher], original copyright [original copyright year].”

 2. If you are unable to obtain permission from the original copyright holder to publish these figures under the CC BY 4.0 license or if the copyright holder’s requirements are incompatible with the CC BY 4.0 license, please either i) remove the figure or ii) supply a replacement figure that complies with the CC BY 4.0 license. Please check copyright information on all replacement figures and update the figure caption with source information. If applicable, please specify in the figure caption text when a figure is similar but not identical to the original image and is therefore for illustrative purposes only. >> We supplied a replacement figure that complies with the CC BY 4.0 license.

4. Please review your reference list to ensure that it is complete and correct. If you have cited papers that have been retracted, please include the rationale for doing so in the manuscript text, or remove these references and replace them with relevant current references. Any changes to the reference list should be mentioned in the rebuttal letter that accompanies your revised manuscript. If you need to cite a retracted article, indicate the article’s retracted status in the References list and also include a citation and full reference for the retraction notice. >> We corrected the reference list as well as the citations in the manuscript. Two references appeared in the reference list by mistake (they were not cited in the text) so we removed them (Barataud 2015 and Krings et al 2022).

Reviewers' comments:

**Comments to the Author**

1. Is the manuscript technically sound, and do the data support the conclusions?

The manuscript must describe a technically sound piece of scientific research with data that supports the conclusions. Experiments must have been conducted rigorously, with appropriate controls, replication, and sample sizes. The conclusions must be drawn appropriately based on the data presented.

Reviewer #1: Yes

Reviewer #2: Yes

Reviewer #3: Yes

2. Has the statistical analysis been performed appropriately and rigorously?

Reviewer #1: Yes

Reviewer #2: N/A

Reviewer #3: Yes

3. Have the authors made all data underlying the findings in their manuscript fully available?

The [PLOS Data policy](http://www.plosone.org/static/policies.action#sharing) requires authors to make all data underlying the findings described in their manuscript fully available without restriction, with rare exception (please refer to the Data Availability Statement in the manuscript PDF file). The data should be provided as part of the manuscript or its supporting information, or deposited to a public repository. For example, in addition to summary statistics, the data points behind means, medians and variance measures should be available. If there are restrictions on publicly sharing data—e.g. participant privacy or use of data from a third party—those must be specified.

Reviewer #1: Yes

Reviewer #2: Yes

Reviewer #3: Yes

4. Is the manuscript presented in an intelligible fashion and written in standard English?

PLOS ONE does not copyedit accepted manuscripts, so the language in submitted articles must be clear, correct, and unambiguous. Any typographical or grammatical errors should be corrected at revision, so please note any specific errors here.

Reviewer #1: Yes

Reviewer #2: Yes

Reviewer #3: Yes

5. Review Comments to the Author

Please use the space provided to explain your answers to the questions above. You may also include additional comments for the author, including concerns about dual publication, research ethics, or publication ethics. (Please upload your review as an attachment if it exceeds 20,000 characters)

Reviewer #1:

1) Suggest removing barotrauma. There are several studies showing barotrauma to be an unlikely cause of mortality in bats. See Lawson et al. 2020, Rollins et al. 2012, and Grodsky et al. 2011. >> Agreed, done.

2) The description of the results and following discussion are rather confusing. I think a figure similar to figure 2, that summarize the actual bat activity would go a long way in simplifying the results. >> We fear that representing the actual bat activity in the same way we represented the predictions could be misleading because of the design, the potential variation sources which cannot be considered in this type of figure and the stochasticity of the raw activity. However, we propose to include information about the raw data by representing (i) the sampled points in the same type of figure as figure 2 (circles) (Figure 2 B) and (ii) the raw activity in histograms for each dataset (Figure S10).

3) The first couple of sentences in the discussion create doubt in the authors assessment of whether their findings are a result of wind speed patterns or sound. Since they did not account for sound in their study, it cannot be ruled out as a potential factor for attraction/avoidance. >> Agreed, we rephrased this part L251-255.

4) Line 249: '...avoidance only at high wind speeds provides clues about the nature of the underlying mechanisms.' However, in lines 216-218 the authors state that bat activity was higher near the turbine under high wind speed conditions. So, its not simply avoidance at high wind speeds. >> Thank you for your comment. By re-reading it, we realized that our phrasing was not clear at all. We meant that avoidance of the turbine was only detected at high wind speed (i.e. not for low wind speed) and not that the only response at high wind speed was avoidance (i.e. not attraction). We rephrased it L266-268 and we hope that it removed any ambiguity.

5) Line 250-251: 'Our findings should considerably improve predictions and thus mitigation of the impacts of wind turbbine on bats.' How? >> By being able to predict more precisely where are the major risks of habitat loss (i.e. downwind the turbine) and thus taking into account both the prevailing wind direction and bat habitats to decide where to implant a wind turbine regarding these parameters. We specified this sentence L268-271.

6) Line 270-271:'At high wind speeds..., we detected more P. pipistrellus activity far away from the wake area than close to it.' This statement contradicts lines 216-218. >> We are sorry for this lack of clarity and we understand where the confusion comes from. We rephrased both parts trying to make clearer whether we are talking about	avoidance of the turbine in terms of distance (i.e. linear gradient) or avoidance of the wake area (i.e. circular gradient) L230, 291 and 294.

7) Starting at line 293 through 297 there is reference to (24). Assuming this is intended to be a reference, perhaps Cryan et al. 2014? >> Yes, thank you very much. We corrected the problem with the references n°24 and n°36.

8) Apologies if I missed this, but I dont recall seeing an explanation for why bats would avoid wind turbines from the windward side under high wind speed conditions. >> You are right. We added the following sentence : « In such disturbed conditions, bats could perceive these turbulent areas at landscape scales as suggested by Cryan et al. 2014, explaining this avoidance of the turbine windward the turbine. » l.296-297.

Reviewer #2: The study relies on a robust dataset from bat passes around the wind turbines in western France. They tested new approaches to how wind turbines affect bat activity, installing autonomous recorders in different places around the turbines to see if the bats are avoiding or are attracted by the turbines, which is innovative and exciting. Although I am a little worried about some statistical analyses used and I highly recommend clarifying some aspects. The introduction is consistent and well-written. The discussion addresses the results found in the study, and it seems that the authors did a good job in the literature review. I miss more figures of the results, and I recommend transforming the contents of the table into graphs to better visualization. >> Thank you for your comment. We completed the statistical analyses part. We transformed the second table into a graph as you recommended. We however kept the first table as we think that figure 2 allows a visualization of its content. Therefore, the table format complete this figure. We also added two more figures (Fig. 2B and Fig. S10) for better vizualisation of raw data.

I have specific concerns during the text, and you can see below:

L88: Fig. 1. (B) The legends are tiny. >> Thank you. We changed the size of the legends, axis titles and axis labels.

L121: I do not know this software and I don’t know how this software is efficient in identifying automatically the bat species. So I recommend citing other published studies that used this software for the automatic identification of the data. >> Thank you for your comment. It’s indeed a software mostly used in France. We added the citation of other published studies as recommended L125.

L121-123: As I am a bat ecologist not european, I suggest talking more about the bat species that you chose. Which is the foraging strategy of this species? Diet and height of the foraging? >> Of course, our apologies. We added some information about the foraging strategy and diet L129-130. The height of foraging is strongly related to the height of the foraging habitat, as *Pipistrellus pipsitrellus* mainly forages at edges, including canopy. Moreover, the data regarding flight height in bats is very scarce. We therefore prefered to not include this last information.

L149: Did you include the sampling nights as a random effect on the models? I saw in the Supplementary Material that you included (1|site) + (1|night) in the full models (Table S4). Why do you include these two variables separated from each other? Did you test if these random effects are the best for the models? >> We indeed included the sampling night and the sampling site in the model including all wind speed to take into account the fact that we sampled various sites each night and each site during a few nights. However, we did not included the sampling night in the models conducted in subsets, as in these models the sampling night explained very little deviance part and led to convergence problems.

L150: I miss the specifications of the family distribution chosen by each model. Did you use “DHARMa” package to check the model residuals as well as the overdispersion and zero inflation? It is common that these datasets of bat activity are overdispersed. >> You are right, we missed specifying this part of the statistical analysis. We now do it l.155-159 « We used a negative binomial error distribution (nbinom1). In each model we performed, we checked that there was no collinearity issues using the check_collinearity function (r package performance), no overdispersion (testDispersion function, r package DHARMa) and we also checked model assumptions by plotting the residuals using the package DHARMa (simulateResiduals function). »

L191-228: I suggest clarifying why you analyzed the AIC models with all variables but included only the GLMM models the distance from the wind turbine, wind incidence angle, and interaction. For example, I saw in Table S4 that mean temperature was a variable that was presented in the dataset of all wind speeds, low and high speed. If these variables were presented in the best models of AIC, it is possible to explain a little bit of the effects? >>We choose not to present results of other variables that the one of interest to avoid too much complexity in the manuscript. However, we understand that it may be missing. We therefore propose to present these results in the supporting information (S6 Table). We now mention this new table L186 in the manuscrit.

Table 1. I recommend transforming the results of the table into a graph/figure. May the coefficient plots or Dot and Whisker plots be formats for these results? >> Thank you for your comment. This seems very relevant for the second table, wich only presents one variable and one dataset. We thus transformed this second table into a figure. We hope it allows a better visibility of the results. However, we fear that transforming the results of the first table into a graph/figure would be confusing as each estimate corresponds to a different variable from different dataset and for two dataset two variables were not retained in the model and thus would not appear in the figure.

L330: Another aspect that is important to mention is the hourly variation of bat activity. The effect of high-speed turbines may be stronger at the beginning of the night when the bat activity is higher. I recommend discussing how this finer temporal scale can affect your results. (May you can test this in a future study?!) >> Thank you for your comment. It is indeed a very relevant suggestion and we would love to test for these effects at a finer temporal scale. It will probably be the object for a future study. In the meanwhile, we add a sentence in the discussion as you suggested L358-360 *« A finer temporal scale could also be considered to test whether bats adapt their foraging or commuting habitat through the night depending on the wind direction and whether the impact of the wake is stronger in hours of high activity*. ».

Reviewer #3: General Comments:
The authors test if the wake effect changed Pipistrellus pipistrellus bat acoustic activity patterns around operating wind turbines in western France in June using acoustic detectors at varying distances from wind turbines. They predicted less bat activity on the leeward side of the wind turbine during wind speeds when wake effect would be moderate to large. They do a thorough statistical analysis of the data and provide adequate discussion of results. This paper contributes to our understanding of how the wake effect could play a role in where bat activity occurs during high-risk periods around wind turbines. It also adds more evidence for attraction to wind turbines. I recommend this manuscript for publication with some improvements, particularly to the discussion. >> Thank you very much for your encouraging comment. We made changes in the manuscript to include all your suggestions.

Discussion:
In lines 243 to 245 it is stated that turbine-generated noise affecting bats has not been tested, but this is not accurate. There are several studies conducted in North America (refer to Guest et al. 2022 and the section on noise) that could be discussed and cited. If the authors mean to say that turbine-generated noise has not been test for this species or in Europe, then the text should be altered to specify. >> That’s true, sorry for that. We changed the sentence to « However, whether the pattern of propagation of turbine-generated noise is the same as the one of the wake distribution patterns remains to be tested and the hypothesis that this noise could affect bats in a way causing either attraction or avoidance seems rather unlikely as the few studies conducted on the topic have shown that potential ultrasonic sounds emitted by a turbine attenuate on short distances (Guest et al. 2022). » L257-261
The discussion lacks any implications on deterrent technology placement, which could strengthen the conclusions. I recommend expanding on this with the findings that during the highest risk periods (when wind turbines are operational at moderate to high wind speeds) bats are most acoustically active on the windward side. This could inform on deterrent placement particularly in areas with a dominant prevailing wind direction. >> You are right. We added a sentence in the discussion L271-273 « These results could also be considered in deterrent technology placement for relatively high wind speed, although this measure efficiency for *P. pipistrellus* remains to be confirmed [40]. »

For future recommendations to further this type of research, is there any reason to believe this pattern would be different in August when peak fatality occurs? I think it would be helpful to include comment here. >> We think that the pattern could indeed be different. However, we have no hypothesis to predict how it could vary. We completed our sentence in the discussion about the pontential variation of the pattern in autumn to include your suggestion L346-351 : « It would be relevant to conduct a similar study in autumn **and in the end of the summer, when peak of bat activity near the turbine (Goldenberg et al. 2021) and peak of fatalities (Baerwald and Barclay, 2011) occur**, to determine whether bats respond to wind turbine wakes in the same way (i) in a migration context and (ii) on windier nights (more windy hours). **Indeed, bat behavior and habitat use can be different in these periods, compared to the reproduction period, involving for example movements along one prevailing direction. Thus, bat sensitivity to the wake effect could vary.** »

Additionally, pairing acoustics with cameras would allow for some assessment of abundance and reduce limitations of acoustic analyses (e.g., is it a single bat versus 100; some bats don’t always echolocate and go undetected acoustically). >> That’s a good idea. We added it in the discussion L358.

General Comments/Edits:
Many brackets are in the wrong location or are the incorrect facing brackets throughout results and tables. Font changes throughout and citation style is not consistent. Suggest a thorough editorial review. >> We reviewed the fonts and size according to Plos One policy. We also corrected the citation style.
Lines 192-195 are restating the methods and can be removed. >> Done.
Lines 195-198-you can’t have negative bat passes so it might be better to state the range instead of the SD here. >> Done.
Line 211-remove colon and replace with “and” >> We think that it would change the meaning. We replace the colon by « as » to be more explicit.
Lines 293-297 there is a switch in citation style to numbers. >> We now reformatted all citations to fit with the Plos One format.
Lines 318-319 seems like a good place for a new paragraph. >> Agreed, done, thank you.

6. PLOS authors have the option to publish the peer review history of their article ([what does this mean?](https://journals.plos.org/plosone/s/editorial-and-peer-review-process#loc-peer-review-history)). If published, this will include your full peer review and any attached files.


If you choose “no”, your identity will remain anonymous but your review may still be made public.


**Do you want your identity to be public for this peer review?** For information about this choice, including consent withdrawal, please see our [Privacy Policy](https://www.plos.org/privacy-policy).

Reviewer #1: No

Reviewer #2: No

Reviewer #3: No

[NOTE: If reviewer comments were submitted as an attachment file, they will be attached to this email and accessible via the submission site. Please log into your account, locate the manuscript record, and check for the action link "View Attachments". If this link does not appear, there are no attachment files.]

While revising your submission, please upload your figure files to the Preflight Analysis and Conversion Engine (PACE) digital diagnostic tool, <https://pacev2.apexcovantage.com/>. PACE helps ensure that figures meet PLOS requirements. To use PACE, you must first register as a user. Registration is free. Then, login and navigate to the UPLOAD tab, where you will find detailed instructions on how to use the tool. If you encounter any issues or have any questions when using PACE, please email PLOS at [figures@plos.org](mailto:figures@plos.org). Please note that Supporting Information files do not need this step.
